# TURNING THE TIDE: PROMISING INITIATIVES IN GEROPSYCHOLOGY TRAINING AND RECRUITMENT

**DOI:** 10.1093/geroni/igz038.2223

**Published:** 2019-11-08

**Authors:** Katherine King

**Affiliations:** William James College, Newton, Massachusetts, United States

## Abstract

The field of geropsychology continues to attract insufficient numbers, and there is a growing sense of urgency among geropsychologists to turn the tide. As a result, a number of new research and programmatic initiatives have been started to better understand the problem and begin to generate solutions. This symposium will report on several such projects, including work from a number of early career geropsychologists. In this symposium, novel theoretical and pedagogical approaches to geropsychology training will be discussed. Graham explores how individuals develop an intention to work with older adults, and describes a new scale grounded in the theory of planned behavior. Dodson and Mlinac discuss bringing quality improvement into geropsychology training at all levels, while King, Rosowsky, and Jolson report on an experiential, intergenerational volunteer program used to demystify work with older adults and facilitate recruitment. Several presentations include the voices of students themselves. King, Rosowsky, and Jolson describe graduate student reactions to volunteering with older adults such as having previous negative beliefs about aging challenged. Bloom-Charette explores the experiences of geropsychology internship applicants, who tend to fare better than generalist applicants and feel well supported. Strong focuses on the problem of attracting individuals into academic geropsychology. In looking at participant responses to a recent webinar series on the topic, it is notable that a large number felt intimidated and overwhelmed by the prospect of entering academia. Taken together, projects in this symposium offer a variety of new directions for future geropsychology research, recruitment, and educational initiatives.

